# Identification of genetic loci that modulate cell proliferation in the adult rostral migratory stream using the expanded panel of BXD mice

**DOI:** 10.1186/1471-2164-15-206

**Published:** 2014-03-19

**Authors:** Anna Poon, Daniel Goldowitz

**Affiliations:** 1 Centre for Molecular Medicine and Therapeutics, Child and Family Research Institute, Department of Medical Genetics, University of British Columbia, Vancouver, BC V5Z 4H4, Canada

**Keywords:** Neural progenitor cells, Adult neurogenesis, Rostral migratory stream, Cell proliferation, Recombinant inbred mice, Quantitative trait locus mapping

## Abstract

**Background:**

Adult neurogenesis, which is the continual production of new neurons in the mature brain, demonstrates the strikingly plastic nature of the nervous system. Adult neural stem cells and their neural precursors, collectively referred to as neural progenitor cells (NPCs), are present in the subgranular zone (SGZ) of the dentate gyrus, the subventricular zone (SVZ), and rostral migratory stream (RMS). In order to harness the potential of NPCs to treat neurodegenerative diseases and brain injuries, it will be important to understand the molecules that regulate NPCs in the adult brain. The genetic basis underlying NPC proliferation is still not fully understood. From our previous quantitative trait locus (QTL) analysis, we had success in using a relatively small reference population of recombinant inbred strains of mice (AXBXA) to identify a genetic region that is significantly correlated with NPC proliferation in the RMS.

**Results:**

In this study, we expanded our initial QTL mapping of RMS proliferation to a far richer genetic resource, the BXD RI mouse strains. A 3-fold difference in the number of proliferative, bromodeoxyuridine (BrdU)-labeled cells was quantified in the adult RMS of 61 BXD RI strains. RMS cell proliferation is highly dependent on the genetic background of the mice with an estimated heritability of 0.58. Genome-wide mapping revealed a significant QTL on chromosome (Chr) 6 and a suggestive QTL on Chr 11 regulating the number of NPCs in the RMS. Composite interval analysis further revealed secondary QTLs on Chr 14 and Chr 18. The loci regulating RMS cell proliferation did not overlap with the suggestive loci modulating cell proliferation in the SGZ. These mapped loci serve as starting points to identify genes important for this process. A subset of candidate genes in this region is associated with cell proliferation and neurogenesis. Interconnectivity of these candidate genes was demonstrated using pathway and transcriptional covariance analyses.

**Conclusions:**

Differences in RMS cell proliferation across the BXD RI strains identifies genetic loci that serve to provide insights into the interplay of underlying genes that may be important for regulating NPC proliferation in the adult mouse brain.

## Background

The persistent division of neural progenitor cells (NPCs) and the production of new neurons in the adult brain raise hope for potential therapies targeting the NPCs to compensate for neuronal loss in injured or disease brains. This process of continual neuron production, also known as adult neurogenesis, occurs in discrete brain regions that include the subgranular zone (SGZ) of the dentate gyrus, the subventricular zone (SVZ) of the lateral ventricle, and the rostral migratory stream (RMS) which is the rostral extension of the SVZ [1,2]. Previous studies have detected increased NPC proliferation under pathological conditions, and the neural precursor cells generated were recruited to the affected brain regions [3-5]. To develop effective strategies that harness the NPCs as a renewable source for repair, it will be necessary to understand how neurogenesis is regulated in the mature brain.

There have been considerable advances in knowledge concerning the regulation of adult neurogenesis over the past two decades [6,7]. Adult neurogenesis is a multifactorial process that encompasses several stages including proliferation, migration, and then the differentiation and survival of new neurons. Each stage is dynamically regulated by both extrinsic and intrinsic factors [7,8]. Regulation at early stages of neurogenesis, most notably the proliferation of NPCs, is especially complex as a wide range of extracellular factors, stimuli, transcription factors, and epigenetic modifiers have been identified [9]. A number of morphogens including Wnt, Notch, Sonic hedgehog, and Ephrins have also been shown to regulate cell proliferation in the adult SVZ [7]. External stimuli such as age, exercise, sleep, and stress have been shown to influence NPC proliferation [10-12]. To control the proliferative behaviour of NPCs, these extrinsic factors must act on an intrinsic system where pro- and anti- proliferative genes are differentially regulated to provide instructions to the NPCs on the appropriate time and frequency to divide. The genetic basis of NPC proliferation, however, is not fully understood.

It has also been previously shown that adult neurogenesis is significantly dependent on the genetic background, and the genetic diversity among inbred strains can serve as a reservoir for gene discovery [13-15]. A wide range of differences in the number of proliferative NPC has been quantified in the RMS of nine inbred strains (including DBA/2 J, C57BL/6 J, A/J) and 27 AXB/BXA recombinant inbred (RI) strains derived from the initial mating of C57BL/6 J and A/J mice [14]. Genome-wide mapping of strain differences in RMS cell proliferation using the AXB/BXA panel led to the identification of a significant quantitative trait locus (QTL) on distal chromosome (Chr) 11 that accounts for ~20% of the phenotypic differences observed among the strains [14]. From this earlier work, we suspected the involvement of more than one locus in regulating NPC proliferation. Therefore, in order to fully appreciate the complexity of this process, we explored a separate genetic reference panel called the BXDs. The BXDs is one of the largest murine mapping panel consists of 80 unique BXD RI strains, which is three times the size of the AXB/BXA resource. The BXD RI strains were derived from an initial mating between C57BL/ 6 J and DBA/2 J that was followed by inbreeding F2 progeny for ≥20 generations. The substantial differences in cell proliferation in the RMS of the BXD RI strains allowed us to detect additional QTLs that modulate NPC proliferation. Functional annotation and gene expression analyses using pre-existing transcriptome data highlighted a subset of candidate genes in the mapped chromosomal regions. Common features shared by these candidate genes include expression in the RMS, functional implication in cell proliferation/cell cycle progression, and participation in signalling pathways important for neurogenesis. These findings provide insights into the dynamic interplay of genetic loci and underlying genes that may modulate NPC proliferation in the adult brain.

## Results

### Significant strain-differences in RMS cell proliferation among the BXDs

**Figure 1:**
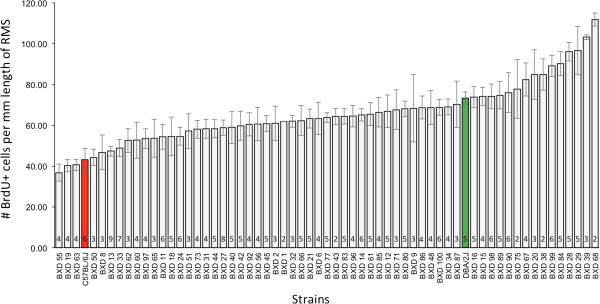
**Quantification of the number of proliferating (BrdU+) cells in the RMS of BXD RI strains.** RMS linear density (i.e. mean number of BrdU + cells per mm length of RMS ± SEM) of 61 BXD RI strains (white bars) and their parental strains, C57BL/6 J (red bar), and DBA/2 J (green bar). The sample size per strain is indicated in the bars.

**Figure 2:**
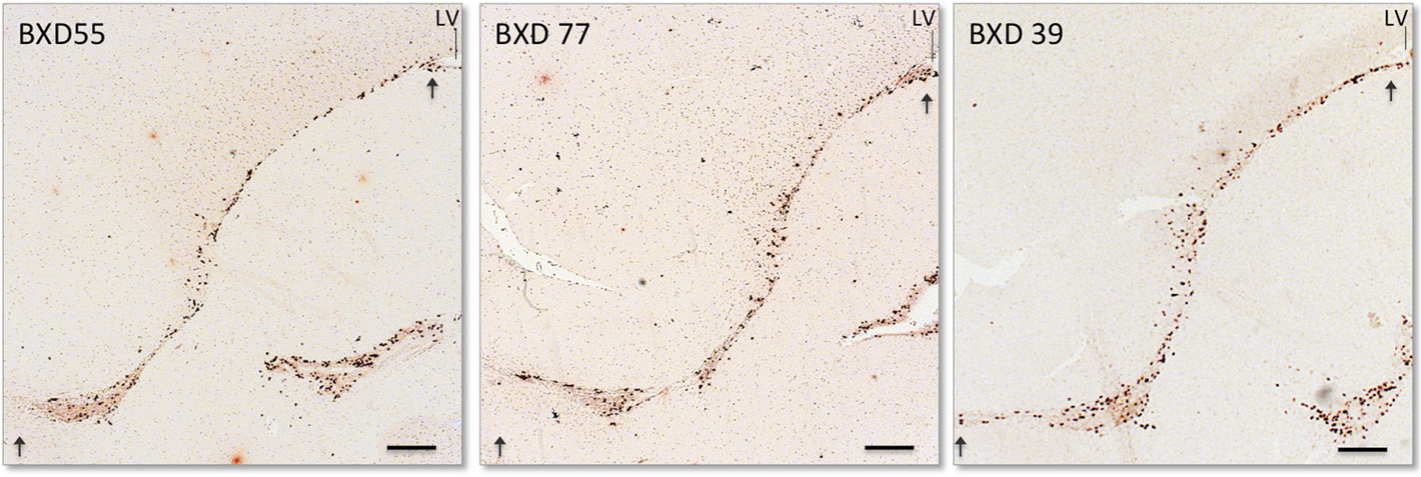
**Representative sagittal sections of BrdU-labeled RMS of three separate BXD RI lines.** All mice received a single pulse of BrdU for one hour. BrdU immunohistochemistry revealed inter-strain differences in BrdU + cell numbers from BXD 39 having high numbers of BrdU + cells in the RMS to BXD 55 having low numbers of BrdU + cells in RMS. Whereas BXD 77 have intermediate numbers of BrdU + cells in the RMS. Arrows mark the beginning and end of the RMS.LV, lateral ventricle; scale bar: 200 μm.

A substantial range of cell proliferation in the RMS, as detected by the uptake of BrdU, was identified in 61 BXD RI strains, with a 3-fold difference among the strains (Figures 1 and 2). This range extends beyond the differences detected between the two parental strains, C57BL/6 J and DBA/2 J (Figure 1). Significant inter-strain differences in RMS linear density was detected (F_60_,_204_ = 4.77 , *P* < 0.0001), and heritability of cell proliferation in the RMS is estimated to be 0.58 (*P* < 0.0001).

There is no significant sex effect (t(263) = 0.82, *P* = 0.4111; females = 63.97 ± 1.66; males = 65.83 ± 1.55) or body weight effect (R^2^ = 0.004; *P* = 0.29) on the number of proliferative cells in the RMS. We also examined the batch effect as the BXD panel has three epoch substructures: i) BXD 1–30 generated by Benjamin A. Taylor in the 1970s [16], ii) BXD 33–42 generated by Benjamin A. Taylor in the late 1990s [17], and iii) BXD 43–103 generated by Peirce and colleagues in the early 2000s [18]. No significant batch effect on RMS linear density was detected (F_2, 262_ = 0.44; *P* = 0.65). However, age had a significant effect on RMS linear density (R^2^ = 0.015; *P* = 0.0442).

We also correlated our RMS linear density data to 3911 traits previously generated using the BXD RI reference panel. RMS linear density (GeneNetwork Trait ID: 13545) is significantly associated with traits from other brain regions such as the hippocampal volume (Trait ID: 10456, r = -0.45, *P* = 0.017), dentate gyrus volume (Trait ID: 10460, r = -0.45, *P* = 0.019), hippocampus granule cell number (Trait ID: 10378, r = -0.46, *P* = 0.017), dorsal striatum volume (Trait ID: 10998, r = -0.30, *P* = 0.04), and striatal neuron number (Trait ID: 13437, r = -0.44, *P* = 0.0006). These phenotypic correlations suggest common genetic determinants underlying RMS cell proliferation and other traits.

### QTLs regulating NPC proliferation in the RMS

**Figure 3:**
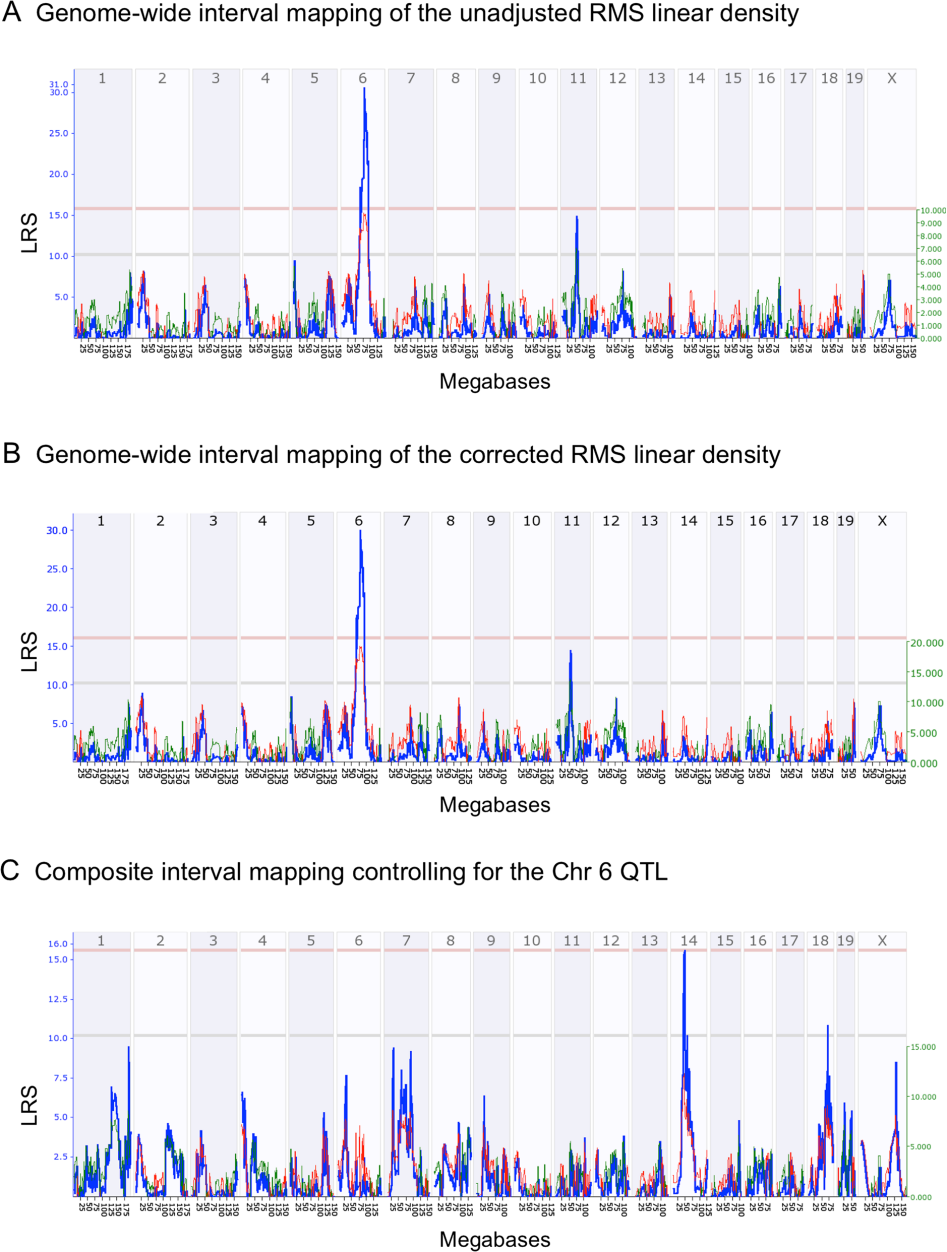
**QTL analyses of cell proliferation in the RMS of BXD RI strains.** The x-axis for figures **(A-C)** represents the chromosomes 1–19, & X (top panel) and their physical maps in megabases (bottom panel). The y-axis and the blue line depict the likelihood ratio statistic (LRS), which indicates the strength of association between genotypes of markers across the genome and the phenotype (i.e. RMS linear density). The light red and gray horizontal lines mark the significant (*P =* 0.05) and suggestive (*P =* 0.63) threshold, respectively. Whole-genome interval mapping of unadjusted RMS linear density **(A)** and for the adjusted RMS linear density corrected for the effects of age **(B)** have mapped a significant QTL on Chr 6 (76.8-88.8 Mb) and a suggestive QTL on Chr 11 (50-58 Mb) regulating RMS linear density. **(C)** Composite interval mapping revealed an additional significant locus on Chr 14 (39–49.5 Mb) and a suggestive locus on Chr 18 (58–86 Mb) that work additively with the Chr 6 QTL in modulating RMS linear density.

To gain insights into the complex genetics regulating adult NPC proliferation, we performed QTL analysis for the differences in RMS linear density among the BXD RI strains. We discovered a highly significant locus on Chr 6 (76.8-88.8 Mb; *P* = 0.05) and a suggestive locus on Chr 11 (50-58 Mb; *P* = 0.63) that modulate NPC numbers in the RMS (Figure 3A). Since age had a significant effect on RMS linear density, we regressed the RMS linear density for each animal against age to ensure the observed genotype-phenotype association is not confounded by differences in age. QTL mapping of the adjusted RMS linear density identified the same significant and suggestive QTLs on Chr 6 and Chr 11, respectively (Figure 3B). Separate QTL analyses were also performed to confirm that mapping results are not confounded by strain epochs. QTL mapping of the old (Taylor) BXD strains and the new (UTHSC) BXD strains both identified the same significant QTL on Chr 6, which again demonstrate the robustness of the Chr 6 QTL (Additional file 1).

We used marker regression analysis to estimate the allelic effect sizes of the mapped QTLs. The genotype of single-nucleotide polymorphism (SNP) and microsatellite markers underlying the Chr 6 QTL revealed that the C57BL/6 J allele is associated with ~19 BrdU + cells/mm increase in RMS cell proliferation compared with having a DBA/2 J allele. Conversely, genetic markers in the Chr 11 QTL region showed that a DBA/2 J allele has an additive effect of ~11 BrdU + cells/mm compared with having a C57BL/6 J allele. These findings suggest the complex genetic modulation of NPC proliferation with the involvement of more than one QTL.

Composite interval analysis revealed secondary intervals on Chr 14 (40.3- 49.2 Mb) and Chr 18 (58.2-74.9 Mb) work additively with the Chr 6 QTL in modulated NPC proliferation in the RMS (Figure 3C). One-way ANOVA confirmed the allelic effects of the Chr 6 (F_1, 58_ = 39.87; *P* < 0.0001), Chr 14 (F_1,58_ = 16.01; *P* = 0.0002), and Chr 18 (F_1, 58_ = 12.56; *P* = 0.0008) on RMS proliferation.

**Figure 4:**
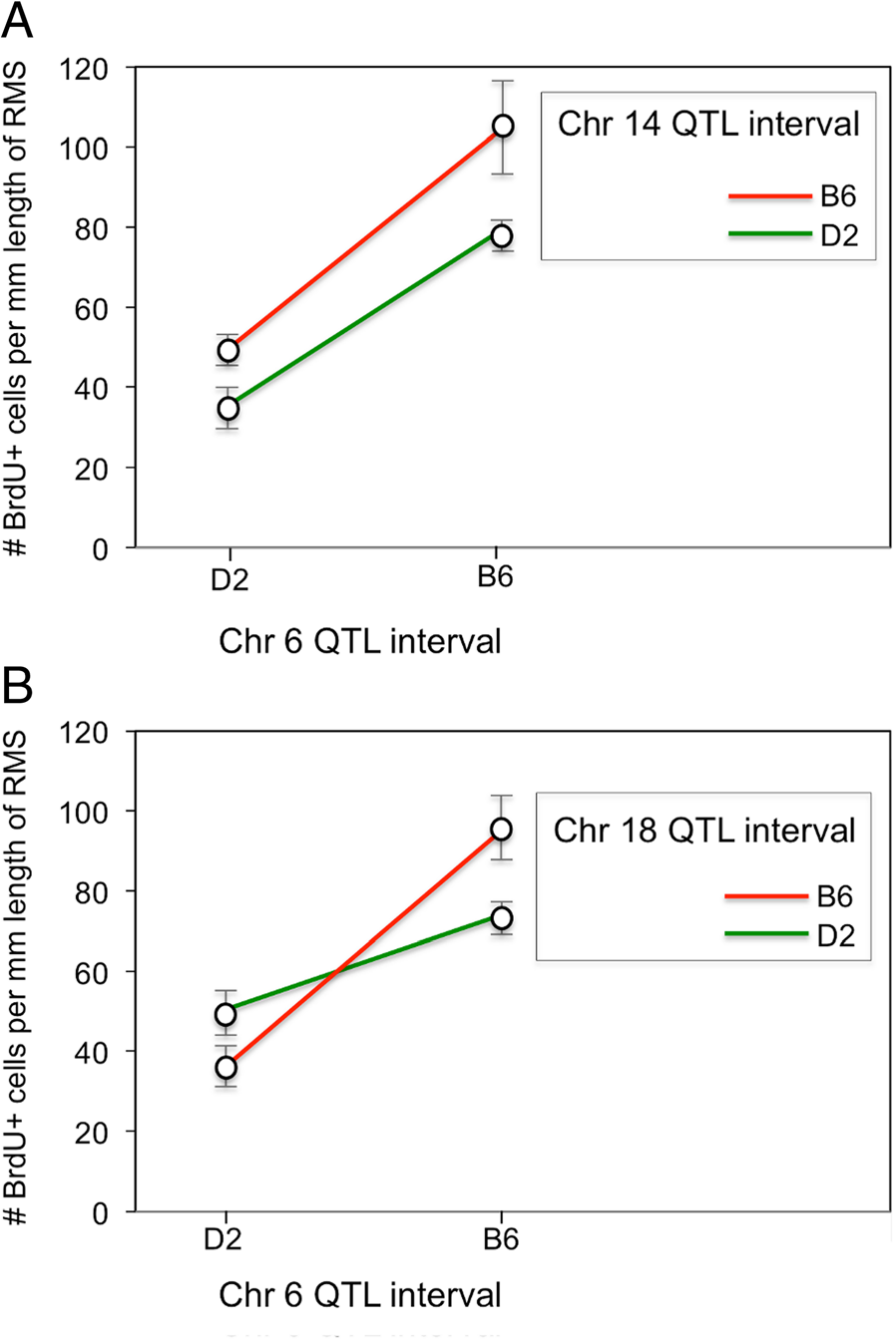
**Allelic effects on cell proliferation in the RMS.** Plots of RMS linear density (i.e. # BrdU + cells per mm length of RMS) versus allele genotypes at markers associated with the major Chr 6 QTLs and secondary QTLs on Chr 14, and Chr 18. BXD strains were divided into different groups based on their genotypes at markers closest to the QTL peaks on Chr 6, 14, and 18. B6 and D2 represent homozygous alleles of C57BL/6 J and DBA/2 J for the markers. Dots represent group means ± SEM. **(A)** Effect of genotype on RMS linear density (y-axis) at markers in the Chr 6 QTL (x-axis) and the Chr 14 QTL intervals (B6 and D2 alleles are represented by red and green colour lines, respectively). **(B)** Effect of genotype on RMS linear density (y-axis) at markers in the Chr 6 QTL (x-axis) and the Chr 18 QTL intervals (B6 and D2 alleles are represented by red and green colour lines, respectively).

Pair-Scan analysis was performed to assess two-way interaction between pairs of genetic markers from different chromosomes. Once again, we found significant association of markers in Chr 6 QTL with markers in the secondary Chr 14 and Chr 18 intervals (LRS Full > 40.1; *P* < 0.01). To further assess the type of interaction among these loci (i.e. additive or epistatic), the BXD strains were split into groups according to their genotypes at these QTL intervals, and an average RMS linear density was calculated for each group. The plotted RMS linear densities show how different allele combinations at these loci are associated with different levels of RMS linear density (Figure 4). The major effect of the Chr 6 QTL is demonstrated where strains carrying the C57BL/6 J allele in the Chr 6 interval are associated with higher RMS linear densities compared to having the DBA/2 J allele, irrespective of the genotypes at the Chr14 or Chr18 QTL intervals. When the genotypes at these secondary QTL regions are taken into account, having the C57BL/6 J allele at either the Chr 14 (Figure 4A) or Chr 18 intervals (Figure 4B) are associated with even higher RMS linear densities compared to having the DBA/2 J alleles. The C57BL/6 J alleles in the Chr 14 and Chr 18 QTL regions are respectively associated with ~15 BrdU + cells/mm and ~10 BrdU + cells/mm increase in RMS cell proliferation compared with having DBA/2 J alleles. These findings suggest the major Chr 6 QTL works additively with loci on Chr 14 and 18 when modulating NPC proliferation in the RMS.

### Mapped loci harbour genes that may serve as modulators of NPC proliferation

We predict the mapped genomic regions harbours genes regulating the number of proliferative NPCs in the RMS. To test our hypothesis, we implemented a combination of bioinformatics tools including QTLminer at GeneNetwork (http://www.genenetwork.org/webqtl/main.py?FormID=qtlminer) and DAVID (http://david.abcc.ncifcrf.gov) to obtain information on genes in the QTL regions. We prioritized genes according to 1) the presence of single-nucleotide polymorphisms (SNPs) and/or insertion/deletions(indels), 2) expression of the gene in the RMS neuroblast transcriptome, 3) presence of transcripts in other neurogenic regions (e.g., SVZ or SGZ) in the adult mouse brain, and 4) association with gene ontology (GO) terms such as neurogenesis, cell cycle, or proliferation. In the significant Chr 6 QTL region (76.8-88.8 Mb) there are 105 genes that have known or predicted functions but only 4 genes met all our criteria (Table 1). Two of these genes, transforming growth factor alpha (Tgfa) and minichromosome maintenance deficient 2 mitotin (Mcm2) have been implicated in adult neurogenesis [19,20]. Pathway analysis showed Tgfa to be involved in the ErbB signalling pathway, which regulates diverse biological processes such as proliferation, differentiation, and survival. Whole genome mapping and composite interval analyses also revealed secondary QTLs influencing cell proliferation in the RMS. To determine whether these suggestive loci interact with the major Chr 6 locus at the molecular level, we further examined the candidate genes residing in the suggestive QTLs. In the suggestive Chr 11 QTL region (50-58 Mb), 93 genes have known/predicted functions and three genes met our candidate gene criteria (Table 1). One of the candidate genes is secreted acidic cysteine rich glycoprotein (Sparc) which has been previously shown to promote cell proliferation in the SGZ of the dentate gyrus [21]. In the Chr 14 QTL region (40.3-49.2 Mb), which was revealed through composite interval mapping, there are 46 genes with known/predicted functions and 2 of these met all the criteria listed above: cyclin-dependent kinase inhibitor 3 (Cdkn3) and glucosamine-phosphate N-acetyltransferase 1 (Gnpnat1). Despite not being directly linked to neurogenesis, both of these genes regulate cell cycle progression [22,23]. In the Chr 18 QTL region (58.2-74.9 Mb), there are 101 genes that have known/predicted functions. Five genes met all of our candidate gene criteria (Table 1). One of these genes, SMAD family member 4 (Smad4) has been directly implicated in adult neurogenesis [24]. Pathway analyses revealed the gene calcium/calmodulin-dependent protein kinase II alpha (Camk2a) in Chr 18 QTL is involved in the ErbB signalling pathway, a pathway also shared by Tgfa in the major Chr 6 QTL. Camk2a also participates in the Wnt signalling pathway. Two other genes Ppp2ca in the suggestive Chr 11 QTL and Smad4 in the Chr 18 QTL are also components of the Wnt signaling machinery. These signalling pathways identified from our analyses have been shown to regulate NPC proliferation in the adult brain [25-28].

**Table 1:** Strong candidate genes identified in the chromosomes (Chr) 6, 11, 14, and 18 QTL intervals

**Gene symbol**	**Gene name**	**Chr**	**Gene location: start (Mb)**	**Gene length (kb)**	**SNPs**	**Indels**	**Gene Ontology (GO) annotation**	**Implicated in SVZ-RMS neurogenesis [ref.]**	**Implicated in SGZ neurogenesis [ref.]**
Tgfa	Transforming growth factor alpha	6	86.145	79.742	6	3	Positive regulation of cell division, negative regulation of apoptosis	✔ [19]	
Anxa4	Annexin A4	6	86.687	56.745	134	7	Cell growth and survival, cell proliferation, carcinogenesis		
Gfpt1	Glutamine fructose-6-phosphate transaminase 1	6	86.993	49.362	48	1	Amino sugar and nucleotide sugar metabolism, cell regeneration		
Mcm2	Minichromosome maintenance deficient 2 mitotin	6	88.833	15.307	3	0	DNA replication initiation, DNA unwinding during replication	✔ [20]	✔ [29]
Ppp2ca	Protein phosphatase 2 (formerly 2A), catalytic subunit, alpha isoform	11	51.912	23.926	1	0	Phosphoprotein phosphatase activity, meiosis, negative control of cell growth and division		
Rad50	RAD50 homolog (S. cerevisiae)	11	53.463	57.801	4	0	DNA repair, homologous recombination, cell cycle		
Sparc	Secreted acidic cysteine rich glycoprotein	11	55.208	25.580	83	5	Response to growth factor stimulus, regulation of cell proliferation		✔ [21]
Cdkn3	Cyclin-dependent kinase inhibitor 3	14	45.692	0.05	3	3	Cell cycle arrest, phosphatase activity		
Gnpnat1	Glucosamine-phosphate N-acetyltransferase 1	14	45.996	12.376	1	0	Amino sugar and nucleotide sugar metabolism, actin dynamics, cell cycle progression		
Camk2a	Calcium/calmodulin-dependent protein kinase II alpha	18	61.085	62.521	0	1	G1/S transition of mitotic cell cycle, neuronal synaptic plasticity		
Seh1l	SEH1-like (S. cerevisiae)	18	67.935	17.718	4	2	Cell division, chromosome segregation, mitosis		
Smad4	Similar to MAD homolog 4 (Drosophila)	18	73.799	64.729	160	74	Cell proliferation, tissue morphogenesis	✔ [24]	
Elac1	elaC homolog 1 (E. coli)	18	73.895	19.442	42	7	tRNA 3*'*-end processing, cell growth and proliferation		
Mapk4	mitogen-activated protein kinase 4	18	74.088	136.463	141	70	Cell cycle, protein amino acid phosphorylation		

*Abbreviations*: *SNPs* single-nucleotide polymorphisms, *indels* insertion/deletions.

### Co-expression of candidate genes further revealed pathway dynamics

**Figure 5:**
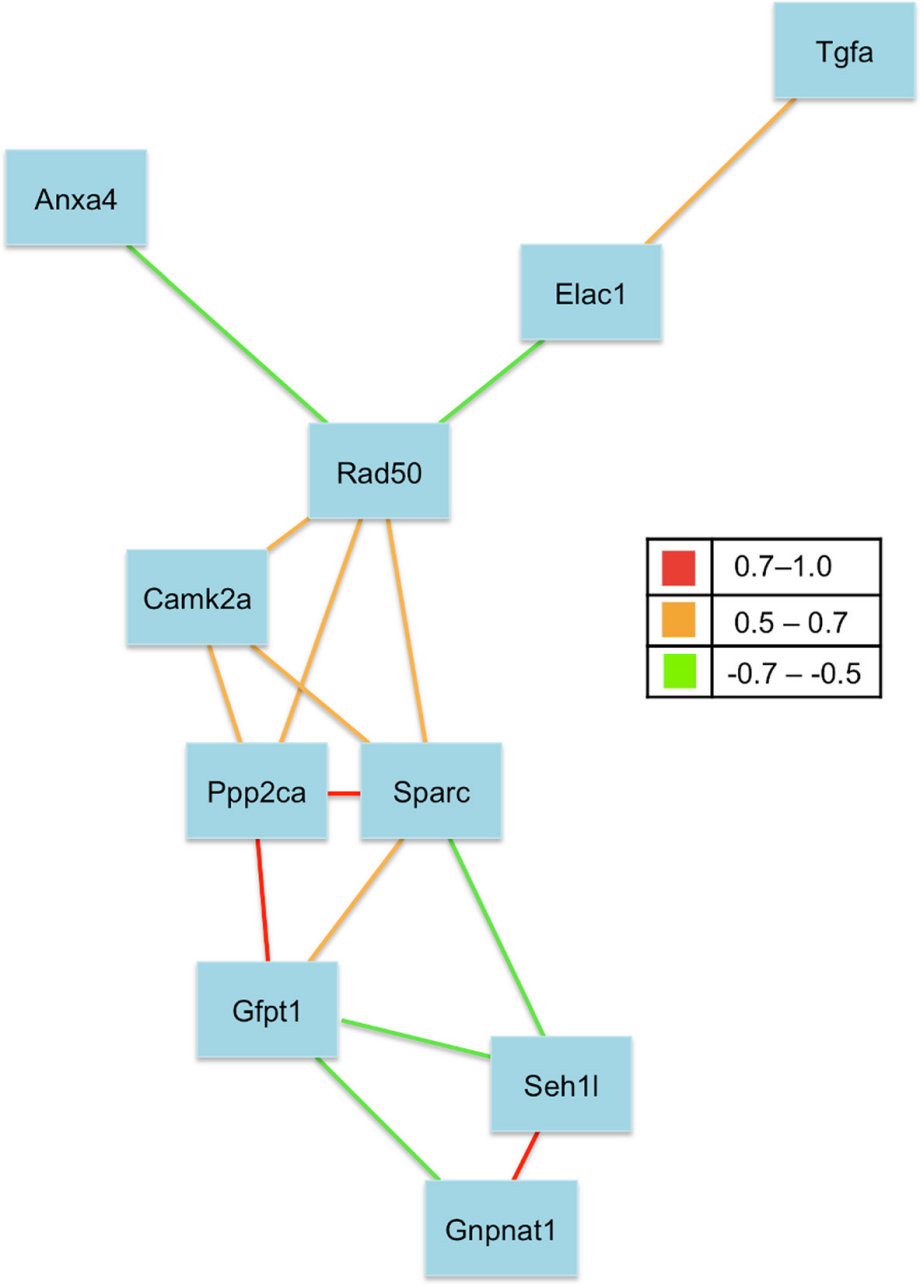
**Transcriptional co-expression network graph of candidate genes.** The transcripts levels of each candidate genes were extracted from the BXD hippocampal expression database available at the GeneNetwork. A network graph was then generated showing how the expression of the candidate genes positively or negatively correlated with each other. Strength of correlation between two connected genes is indicated in the legend.

We further explored the interconnectivity of the candidate genes based on their expression level in the adult brain. We suspect polymorphisms in these candidate genes are likely to affect gene expression level, similar to the heritable differences in number of RMS proliferative cells observed among the BXD. Since there is no RMS transcriptome data available for the BXDs, we used the BXD hippocampal microarray data available at the GeneNetwork [Hippocampus Consortium M430v2 (Jun06) PDNN] to investigate expression co-variation among our candidate genes. The hippocampal transcriptome was selected based on findings from our earlier phenotypic correlation analysis where cell proliferation in the RMS is significantly associated with several hippocampal traits including hippocampus and dentate gyrus volumes and dentate gyrus granule cell number. The transcripts levels of each candidate genes were extracted from the BXD hippocampal expression database. A network graph (Figure 5) was then generated showing how the expression of the candidate genes residing in different loci positively or negatively correlated with each other. We found genes participating in cell proliferation exhibited similar expression levels where Gfpt1 (a gene in the significant Chr 6 QTL) is highly correlated with the expression of two other candidate genes, Sparc (a gene in the Chr 11 QTL; r = 0.767; *P* < 1.00E^-16^) and Ppp2ca (a candidate gene in the suggestive Chr 11 QTL; r = 0.706; *P* = 4.29E^-13^). Ppp2ca, which is involved in the Wnt signalling pathway, is also significantly correlated with Camk2a in the Chr 18 QTL (r = 0.66; *P* = 6.26E^-11^). This finding is consistent with the general assumption that genes in the same pathway exhibit similar gene expression profiles [30]. In addition to gene expression covariance, we also detected significant correlation between gene expression and the phenotype (i.e. RMS linear density). The expression of candidate genes Anxa4 (Chr 6 QTL), Ppp2ca (Chr 11 QTL), Gnpnat1 (Chr14 QTL), and Camk2a (Chr18 QTL) varied across the different BXD strains, and their transcriptional differences are significantly correlated with the phenotypic differences in RMS linear density observed among the BXDs (r = -0.58, r = -.50, r = -0.28, r = 0.29, respectively; *P* < 0.03; N = 56 BXD RI strains; Additional file 2).

### Cell proliferation in the SGZ is regulated by a different set of QTLs

**Figure 6:**
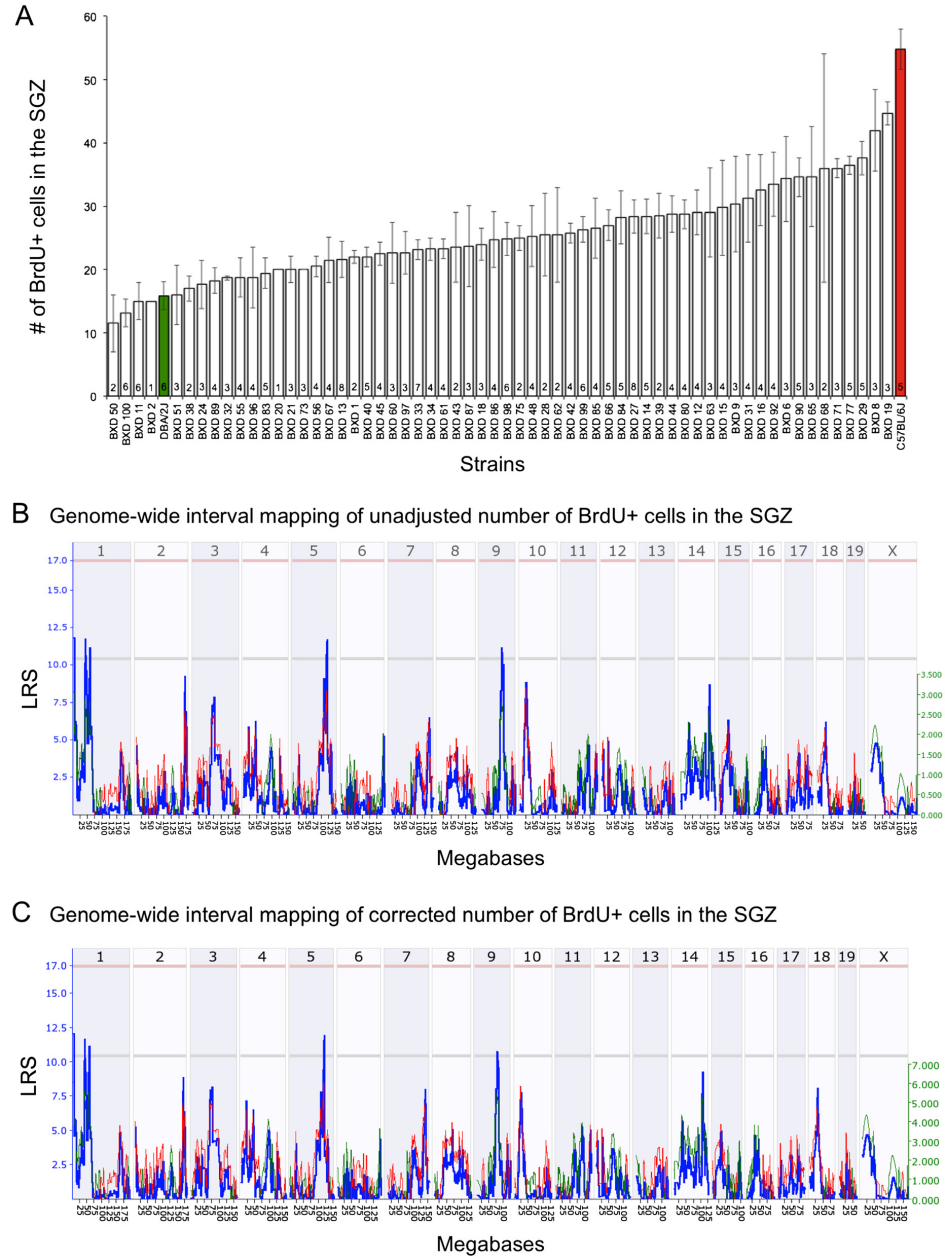
**Cell proliferation in the SGZ of BXD RI strains and QTL analyses. (A)** The number of BrdU^+^ cells in the SGZ (± SEM) of 61 BXD RI strains (white bars) and their parental strains, C57BL/6 J (red bar), and DBA/2 J (green bar). The sample size per strain is indicated in the bars. Whole-genome scan LRS plot generated from QTL mapping of the unadjusted SGZ cell proliferation data **(B)** and the adjusted data corrected for age effects **(C)**. The x-axis represents the chromosomes 1–19, & X (top panel) and their physical maps in megabases (bottom panel). The y-axis and the blue line depict the LRS, which indicates the strength of association between genotypes of markers across the genome and the phenotype. The light red and gray horizontal lines mark the significant (*P =* 0.05) and suggestive (*P =* 0.63) threshold, respectively. Whole-genome interval mapping of unadjusted SGZ proliferation data **(B)** and the SGZ proliferation data adjusted for age **(C)** identified no significant QTL but revealed suggestive QTLs on Chr 1 (40-59 Mb), Chr 5 (104.5-119.5 Mb), and Chr 9 (71–83.7 Mb).

The high correlation between the RMS linear density and the hippocampal traits such as volume and cell numbers, which harbours the SGZ of the dentate gyrus, led us to suspect common genetic determinants underlying these two neurogenic sites. We first assessed cell proliferation in the SGZ one-hour post BrdU injection. Similar to what was observed in the RMS, the number of BrdU + cells in the SGZ significantly differs among the BXD RI strains (F_60_,_170_ = 2.88, *P* < 0.0001) (Figure 6A). Heritability of cell proliferation in the SGZ is estimated to be 0.5 (*P* < 0.0001). The number of BrdU + cells in the SGZ was 3.5 fold higher in C57BL/6 J compared to DBA/2 J, which was opposite to the parental strain differences observed in the RMS. However this reversal in phenotypic direction was not observed in the BXD RI panel. For example, BXD68 had both high numbers of BrdU + cells in the RMS and SGZ. Whereas, BXD55 had low numbers of BrdU + cells in the RMS and SGZ.

The effects of confounding variables on cell proliferation in the SGZ were also examined. Age had a significant effect on the number of BrdU + cells in the SGZ (R^2^ = 0.059, *P* = 0.0002). Strain epoch, sex, body weight differences did not significantly influence the number of proliferative cells in the SGZ.

QTL mapping of cell proliferation in the adult SGZ revealed suggestive QTLs on Chr 1 (40-59 Mb), Chr 5 (104.5-119.5 Mb), and Chr 9 (71–83.7 Mb) and no significant QTL was identified (Figure 6B). QTL mapping of SGZ proliferation corrected for differences in age revealed the same suggestive QTLs on Chr 1, 5, and 9 (Figure 6C). These SGZ QTLs do not correspond to the loci associated with cell proliferation in the RMS and therefore suggest a different set of genetic modulators regulating the number of proliferative cells in the SGZ. This is further supported by the lack of phenotypic correlation between cell proliferation in the SGZ and RMS (r = 0.14, *P* = 0.2917, N = 61 BXD strains).

## Discussion

Adult neurogenesis is a multi-stage process that is complex in terms of regulation as each stage is modulated by different genetic and environmental factors [7,9]. The earliest phase of adult neurogenesis is the proliferation of the NPCs in the adult SGZ, SVZ, and RMS. NPC proliferation is highly heritable and dependent on the genetic background. The effects of genetic variations on NPC proliferation is likely through a network of genes which will be difficult to reveal from conventional single gene study. Here, we used a systems genetics approach where we correlate genetic variation and expression data to NPC proliferation in the adult mouse brain. We took advantage of the genetic and phenotypic diversity in the BXD reference panel, and through genome-wide QTL mapping, we discovered novel genetic loci that likely house modulators of NPC proliferation in the RMS. A closer examination of candidate genes in the mapped loci (using public databases on gene ontology, genetic polymorphisms, and gene expression) identified a subset of genes associated with adult neurogenesis and/or cell proliferation. We further demonstrated interconnectivity among some of these candidate genes by showing they co-vary at the transcriptional level and participate in the same pathways previously shown to control adult neurogenesis.

To probe the genetic architecture of a polygenic phenotype as complex as NPC proliferation requires a large genetic reference panel that can provide high resolution and power to identify genes with subtle but significant effects on NPC proliferation. In this study, we utilized the genetic diversity among the BXD RI strains as a tool to identify genes and pathways involved in NPC proliferation. Our BXD panel of 61 strains is the largest mapping panel employed thus far to study adult neurogenesis, and it is over twice the size the AXB/BXA RI set we have previously examined [14]. In addition, over half of the BXDs in our panel (34 out of 61 strains) were from the more recent UTHSC BXD set, which have approximately twice the number of recombinations per strain compared to the older BXD subset produced by Taylor. These features of our BXD panel improved the statistical power to map QTLs at high resolution as well as uncover loci-loci interaction. Normal distribution of the RMS linear densities in the BXD genetic reference panel indicates that more than one genetic locus is involved in NPC proliferation. Subsequent genome-wide interval mapping provided novel insights into the genetic regions regulating NPC proliferation. A significant Chr 6 QTL was identified to account for 19% of the inter-strain differences, and this locus was found to interact additively with two other genomic regions on Chr 14 and Chr 18. Pairwise interaction between Chr 6 QTL and these secondary loci explained ~40% of the inter-strain differences. Genome-wide mapping also revealed a suggestive locus on Chr 11 that accounts for ~11% of the phenotypic variance in the BXD population and it does not directly interact with the Chr 6 QTL. The identification of interacting and non-interacting genetic loci suggests the genetic network modulating NPC proliferation is comprised of regulatory pathways of variable interactivity.

To better understand the regulatory pathways modulating NPC proliferation in the RMS, we needed to first identify the molecular players participating in these pathways. It is presumed that the mapped loci harbor genetic modifiers of NPC proliferation. Our rather stringent candidate gene analyses revealed several genes in the significant Chr 6 QTL regions that are associated with neurogenesis and cell proliferation. One of them is Tgfa, which is a growth factor that binds to the epidermal growth factor receptors (EGFR). Upon binding to its receptor, Tgfa activates the ErbB signalling pathway and subsequently triggers the Mitogen-activated protein kinase (MAPK) signalling cascade. These pathways play crucial roles in regulating cell proliferation and differentiation. The absence of Tgfa resulted in attenuation of NPC proliferation in the dorsolateral corner of lateral ventricles and reduction of newborn neurons in the OB [19]. Another candidate gene implicated in adult neurogenesis is Mcm2. This gene encodes for a component of the DNA replication machinery for the S phase of cell cycle. Mcm2 has been used as a proliferation marker in adult SVZ and hippocampal neurogenesis [29,31]. Hypomorphic expression of Mcm2 resulted in decreased NPC proliferation in the SVZ [20]. In addition to genes in the Chr 6 region, genes in the suggestive QTL regions also harbour promising candidates that have been previously implicated in adult neurogenesis. One of which is Sparc in the Chr 11 QTL. Sparc encodes a cysteine-rich acidic matrix-associated protein, which is involved in extracellular matrix synthesis, regulation of cell shape, and cellular response to growth factor stimulus. Sparc also affects hippocampal neurogenesis with the knockout of Sparc resulting in decreased cell proliferation in the SGZ of the dentate gyrus [21]. Smad4 in the suggestive Chr 18 QTL has been directly implicated in adult neurogenesis [24]. Smad4 encodes for a signal transduction protein that participates in the Bone morphogenetic protein signaling pathway, Wnt signaling pathway, and the Transforming growth factor beta (TGF-beta) signaling pathway. Conditional knockout of Smad4 in the SVZ neural stem cells greatly impaired neuron production in the OB and induced ectopic oligodendrocytes in the corpus callosum [24].

Candidate gene pathway analysis further provided insights into the interconnection among the candidate genes. Both the Tgfa gene in the major Chr 6 QTL and the Camk2a gene in the secondary Chr 18 QTL are involved in the ErbB signalling pathway, which regulates diverse biological processes such as proliferation, differentiation, and survival. Tgfa serves as one of the extracellular ligands that bind to Epidermal growth factor receptors and subsequently triggers downstream intracellular signalling pathways, which is modulated by Camk2a. Camk2a also participates in the Wnt signalling pathway as well as Ppp2ca in the suggestive Chr 11 QTL. Ppp2ca is one of the major Ser/Thr phosphatases which has been previously implicated as a negative regulator of cell proliferation. Ppp2ca interacts with Axin, a scaffold protein that down-regulates the Wnt signalling pathway by destabilizing β-catenin [32]. These findings highlight the complexity of NPC regulation where genes at different loci converge on the same regulatory pathway and possibly exert their effects in an additive manner. Overlap in genes participating in seemingly independent pathways such as ErbB signalling pathway and Wnt signalling pathways further suggests crosstalk between these pathways. Previous reviews have described the regulation of adult neurogenesis as a fine balance of the different systems of networks [9,33]. The interconnectedness between pathways indicates that the perturbation of one may trigger compensatory changes at others [9].

The interconnectivity among candidate genes is further reflected at the transcriptional level. Because there is no BXD transcriptome data available for the RMS, we probed the hippocampal gene expression database for the transcript levels of our candidate genes. We later confirmed the expression of these candidate genes in the mouse RMS using the microarray data of laser microdissected RMS generated by Khodosevich et al. [34]. Our gene co-expression network indicates the candidate genes glutamine fructose-6-phosphate transaminase 1 (Gfpt1) in the significant Chr 6 QTL as a highly-connected node that positively co-vary with Ppp2ca and Sparc in the suggestive Chr 11 QTL. Gfpt1 also negatively co-varies Gnpnat1 in the Chr 14 QTL and Seh1l in the Chr 18 QTL. Gfpt1 is a rate-limiting enzyme of the hexosamine pathway, and despite not directly implicated in neurogenesis, studies have shown Gfpt1 to positively regulate cell proliferation and regeneration of other tissues [35,36]. The Gfpt1 regulated-hexosamine biosynthesis pathway has been shown to drive the levels of β-catenin and cell proliferation [37]. The expression of β-catenin is the downstream target of the canonical Wnt pathway (or Wnt/β-catenin pathway), which is regulated by other QTL candidate genes such as Ppp2ca. These findings demonstrate the dynamic interplay of candidate genes and the convergence of different pathways to influence cell proliferation in the adult brain.

The high correlation between cell proliferation in the RMS and several hippocampal traits such as the number of granule cells in the dentate gyrus led us to suspect shared genetic determinants underlying proliferation in RMS and dentate gyrus. We examined the QTLs modulating cell proliferation in SGZ of the dentate gyrus using our expanded panel of BXDs. The number of proliferative cells in the SGZ differed ~4 fold among the 61 BXD RI strains. A similar fold difference was reported by Kempermann and colleagues where they used Ki67 as a proliferative marker and quantified the number of dividing cells in the SGZ of 29 BXD RI strains [13]. The proliferative differences we detected among the BXDs were mapped to three suggestive QTLs on Chr 1, Chr 5, and Chr 9. These loci did not overlap with the QTLs regulating RMS cell proliferation. From these findings we concluded that there are separate sets of genes that differentially modulate NPC proliferation at the RMS and SGZ.

## Conclusions

The bottom-up approach, starting with the manipulation of a single gene to induce changes in phenotype, has yielded valuable insights into a gene’s role in adult neurogenesis. However, this approach is limited in capturing the full complexity of adult neurogenesis where several genes and signalling pathways can regulate neurogenesis in the adult brain. In this study, we gained insights into the genetic architecture underlying NPC proliferation using a top-down, phenotypic-driven approach.

The rich genetic and phenotypic diversity in the expanded panel of BXD RI strains has allowed us to probe the complex networks regulating NPC proliferation in the adult mouse brain. Here, we performed genome-wide scans for QTLs associated with the differences in the number of NPCs among the BXD RI strains. Novel QTLs and significant loci-loci interaction were detected. Using bioinformatics resources and transcriptome databases, we further identified several candidate genes with correlating expression patterns and discovered the convergence of these genes on the same/parallel signalling pathways that are known to regulate adult neurogenesis. The combinatorial influence of these regulators on NPC proliferation remains to be elucidated. Nevertheless, our findings provide a firm starting point to unravel modifier genes of NPC proliferation and offer novel insights into the dynamic interplay of regulatory pathways controlling this process.

## Methods

### Animals

BXD RI strains were obtained from two different sources. Parental strains C57BL/6 J, DBA/2 J, and BXD 1–42 RI strains were purchased from the Jackson Laboratory (Bar Harbor, ME, USA). BXD 43–103 RI strains were provided by Dr. Robert W. Williams and Dr. Lu Lu (University of Tennessee Health Science Center, Memphis, TN, USA). Mice were housed at the University of Tennessee Animal Facility in a pathogen-free, ~23.5°C, and 45–50 humidity environment on a 12 h light-12 h dark cycle. A total of 61 BXD RI strains and 265 mice were used in this study. At least one male and one female per BXD strain were examined. Mice studied were between 50–85 days old. All experiments were conducted according to the Guide for the Care and Use of Laboratory Animals (National Institutes of Health, USA) and the Canadian Council of Animal Care. Approval was obtained from the Institutional Animal Care and Use Committee at the University of Tennessee Health Science Center and the Animal Care Committee at the University of British Columbia on all our animal protocols.

### BrdU administration and detection

All mice received a single intraperitoneal injection of BrdU (50 mg BrdU kg^-1^) to label actively proliferating cells in their brains. One-hour post injection, mice were perfused with acetic acid: 95% ethanol (1:3) as previously described [14]. Brains were collected and paraffin embedded. Embedded brains were sagittally sectioned at 8 μm and every 10^th^ section was mounted on Superfrost/Plus slides for anti-BrdU immunohistochemistry. In brief, sections were deparaffinzed, and treated with 1 M HCl for 30 mins at 37°C to denature DNA. Slides were incubated with the mouse primary anti-BrdU monoclonal antibody (1:200; BD Biosciences, Mississauga, ON, Canada) overnight at room temperature. The next day, secondary biotinylated horse anti-mouse IgG (1:200, Vector Laboratories, Burlingame, CA, USA) was applied to the slides for 1 h at room temperature VECTASTAIN Elite ABC kit (Vector Laboratories) and 3, 3′-diaminobenzidine (DAB; Sigma-Aldrich) were subsequently used to reveal BrdU immunoreactivity.

### Quantification

The number of proliferative (BrdU+) cells in the RMS was systematically quantified using the single best-section quantification method as previously described [14]. Briefly, sagittal sections encompassing the medial to lateral extend of the RMS of the left hemisphere was examined, and the section containing the RMS exhibiting the fullest extent of the SVZ-OB trajectory was identified. The number of BrdU + cells in the RMS of this optimal section was quantified under brightfield illumination (20× objective; Zeiss 200 M Axiovert inverted microscope equipped with Axiovision 4.6 software). Images of the quantified RMS were captured using the same inverted microscope, and the length of the RMS was subsequently measured with NIH ImageJ (version 1.42) software. For each animal, the RMS linear density, which is the number of BrdU + cells per mm of RMS length, was calculated. The average RMS linear density and standard error of the mean (SEM) were determined for each strain. To demonstrate the effectiveness of our single best-section quantification method, we also quantified the total number of NPCs in the RMS of 20 randomly selected RI mice where we counted the number of BrdU + cells in every 10^th^ section throughout the entire medial to lateral extent of the RMS. We found the total NPC counts were highly correlated with the RMS linear densities of these animals (R^2^ = 0.7744; *P* < 0.0001; Additional file 3).

Mice analyzed for RMS cell proliferation were also used to quantify cell proliferation in another active site of adult neurogenesis called the SGZ. The SGZ is located between the granular layer and hilus of the dentate gyrus of the hippocampus. The number of proliferative, BrdU + cells in the SGZ was counted throughout the left dorsal hippocampus for every tenth sagittal section. Counts stopped where the dorsal and ventral components of the hippocampus merged. The average number of BrdU + cells in the SGZ and SEM were determined for each BXD strain.

### Statistical analysis

One-way analysis of variance (ANOVA) was performed to detect significant inter-strain differences in the number of proliferative cells in the RMS (JMP 10 statistical software, SAS Institute, Cary, NC, USA). Tukey’s Honestly Significant Difference (HSD) post-hoc test was subsequenlty employed to determine signficant differences between two BXD strains. General linear modeling was used to determine the contribution of possible confounding covariates including age, sex, body weight, and strain epoch effects and their interaction effects on RMS proliferation. Residuals from regression fitting these variables for RMS linear density were obtained and subsequently used to adjust for the mean RMS linear density per strain [14]. Analyses that yielded *P* ≤ 0.05 were considered significant. Heritability was estimated in a broad sense where we calcualted the ratio of variance that is accounted for by the differences between strains over the total variance, which includes both between-strain variance and within-strain variance [13].

### QTL mapping

Cell proliferation data collected from the 61 BXD RI strains was deposited into the GeneNetwork, which is an open-access online database that contains detailed genotype information of each BXD RI strain. Genome-wide interval mapping of QTLs regulating NPC proliferation was performed using WebQTL, a module of the GeneNetwork. The likelihood ratio statistic (LRS) was computed to assess the strength of genotype-phenotype association of the genome scans. Permutation test of 2000 permutations was computed to establish the significance and suggestive thresholds where the LRS values corresponded to a genome-wide *P* value of 0.05 and 0.63, respectively. A significant QTL is referred to as a chromosomal region with LRS score equal or above the genome-wide significant level (*P* = 0.05). A suggestive QTL is a region of the chromosome with LRS score equal or above the genome-wide suggestive level (*P* = 0.63). LRS scores of the mapped QTLs were converted to the likelihood of the odds (LOD) scores by dividing LRS by 4.61. The confidence limits of each QTL were defined by the 1.5 LOD support interval [38].

### Candidate gene analysis

An integration of bioinformatics strategies and gene expression data were employed to evaluate the underlying genes in the mapped QTL intervals. The genetic variation structure within identified QTL regions were examined using the single-nucleotide polymorphism (SNP) and insertion/deletion (indel) data available at the GeneNetwork SNP browser (genenetwork.org/webqtl/snpBrowser.py). The numbers of SNPs and indels that are associated with each candidate gene, and ones that differ between the two parental inbred strains (i.e., DBA/2 J and C57BL/6 J) were determined. Sequencing data released by the Mouse Genomes Project (http://www.sanger.ac.uk/resources/mouse/genomes/) was used to confirm the presences of SNPs and indels in each of the candidate gene. The expression of each candidate gene in the adult brain is visualized using Allen Brain Atlas (http://www.brain-map.org). Microarray data on laser-microdissected NPCs in the RMS [34] was used to determine the presence/absence and transcript level of a candidate gene in the RMS. Candidate gene were further assessed on their associated Gene Ontoloy (GO) terms and pathways using information available at the Database for Annotation, Visualization, and Integrated Discovery (DAVID, http://david.abcc.ncifcrf.gov) and the QTLMiner module of the GeneNetwork (http://www.genenetwork.org/webqtl/main.py?FormID=qtlminer). Genes were prioritized according to the following criteria: 1) polymorphic (i.e. associated with ≥ 1 SNPs and/or Indels), 2) expression in the adult neurogenic sites including the RMS, and 3) associated with GO terms such as neurogenesis, cell proliferation, and cell cycle progression.

### Availability of supporting data

BXD data supporting the results of this article are available in the BXD Published Phenotypes Database at the GeneNetwork (http://www.genenetwork.org) under Record IDs: 13545 (RMS linear density, unadjusted data), 13586 (RMS linear density data corrected for age effects), 14786 (SGZ BrdU + cell counts, unadjusted data), and 16187 (SGZ BrdU + cell counts corrected for age effects).

Other data supporting the results of this article are included as additional files (Additional files 1, 2, and 3) within the article.

## Abbreviations

BrdU: Bromodeoxyuridine; Chr: Chromosome; NPCs: Neural progenitor cells; RI: Recombinant inbred; RMS: Rostral migratory stream; SGZ: Subgranular zone; SVZ: Subventricular zone; QTL: Quantitative trait loci.

## Competing interests

The authors declare that they have no competing interests.

## Authors’ contributions

DG conceived the study. AP collected phenotypic and carried out all data analyses including QTL mapping and candidate gene analysis. AP and DG wrote the manuscript. Both authors read and approved the final manuscript.

## Supplementary Material

Additional file 1 **Whole-genome QTL analyses of the old and new BXD sub-populations.** (A) Whole-genome interval mapping of the old BXD strains 1–42 generated by Benjamin A. Taylor; 27 of the 61 BXD strains examined in this study belong to this group (B) Whole-genome interval mapping of the new BXD strains 43–100 generated at the University of Tennessee Health Science Center (UTHSC); 34 of the 61 BXD strains examined in this study belong to this group. Despite differences in LRS scores, the same Chr 6 QTL (76.8-88.8 Mb) is identified from mapping the two BXD sub-populations. Click here for file

Additional file 2 **Correlation between the transcript levels of candidate genes and RMS linear density.** Inter-strain differences in the expression of Anxa4 (a candidate gene in the significant Chr 6 QTL interval), Ppp2ca (a candidate gene in the suggestive Chr 11 QTL interval), Gnpnat1 (a candidate gene in the suggestive Chr 14 QTL interval), and Camk2a (a candidate gene in the suggestive Chr 18 QTL) were observed in the hippocampi of different BXD RI strains. (A) Scatterplot of the Anxa4 transcript levels negatively correlated with the mean RMS linear density. (B) Scatterplot of the Ppp2ca transcript levels negatively correlated with the mean RMS linear density. (C) Scatterplot of the Gnpnat1 transcript levels negatively correlated with the mean RMS linear density. (D) Scatterplot of the Camk2a transcript levels positively correlated with the mean RMS linear density. Each dot represents a BXD strain. Click here for file

Additional file 3 **Correlation between the RMS linear density and total number of proliferating NPCs in the RMS.** RMS linear density (i.e. the number of BrdU + cells per mm length of RMS; x-axis) was determined using the single best-section quantification method, and it is significantly correlated with the total BrdU + cell counts (y-axis) which was determined from surveying every 10^th^ section throughout the medial to lateral extent of the RMS (*P* < 0.0001). Each data point represents counts obtained from a randomly selected RI mouse. Click here for file
