# Supplementary figures and images for: Identification of genetic loci that modulate cell proliferation in the adult rostral migratory stream using the expanded panel of BXD mice
Source: BMC Genomics. 2014 Mar 19;15:206. doi: 10.1186/1471-2164-15-206 (PMC4004255; doi:10.1186/1471-2164-15-206)

# A

## RMS linear density of the Taylor strains BXD 1-42

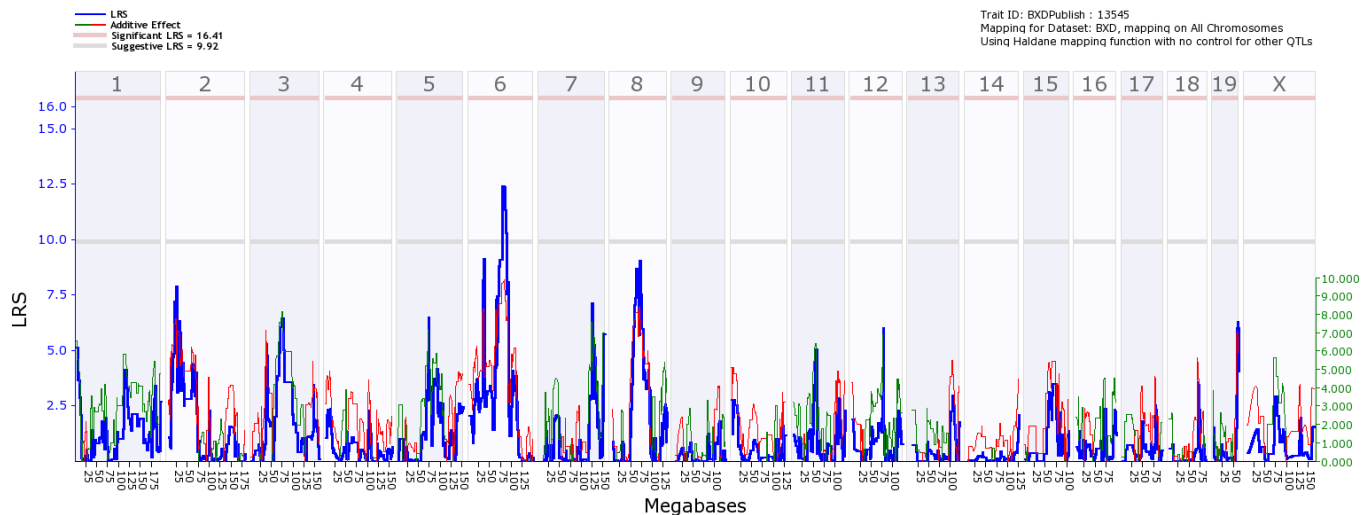

# B

## RMS linear density of the UTHSC strains BXD 43- 100

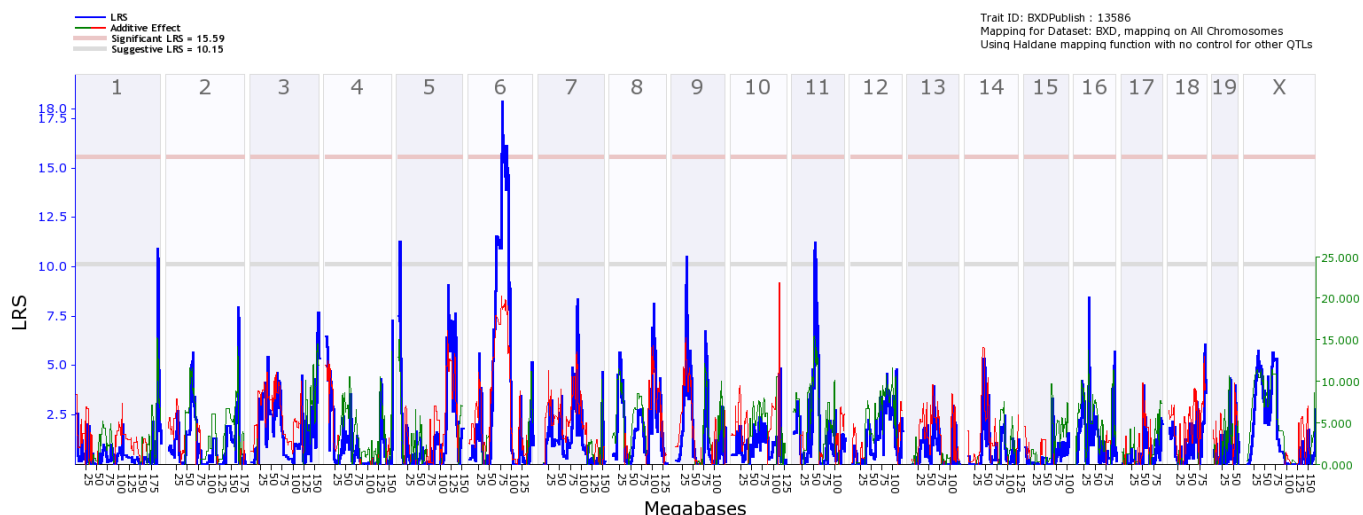

Supplement: Additional file 1 — Whole-genome QTL analyses of the old and new BXD sub-populations. (A) Whole-genome interval mapping of the old BXD strains 1–42 generated by Benjamin A. Taylor; 27 of the 61 BXD strains examined in this study belong to this group (B) Whole-genome interval mapping of the new BXD strains 43–100 generated at the University of Tennessee Health Science Center (UTHSC); 34 of the 61 BXD strains examined in this study belong to this group. Despite differences in LRS scores, the same Chr 6 QTL (76.8-88.8 Mb) is identified from mapping the two BXD sub-populations. [file 1471-2164-15-206-S1.pdf]

**A**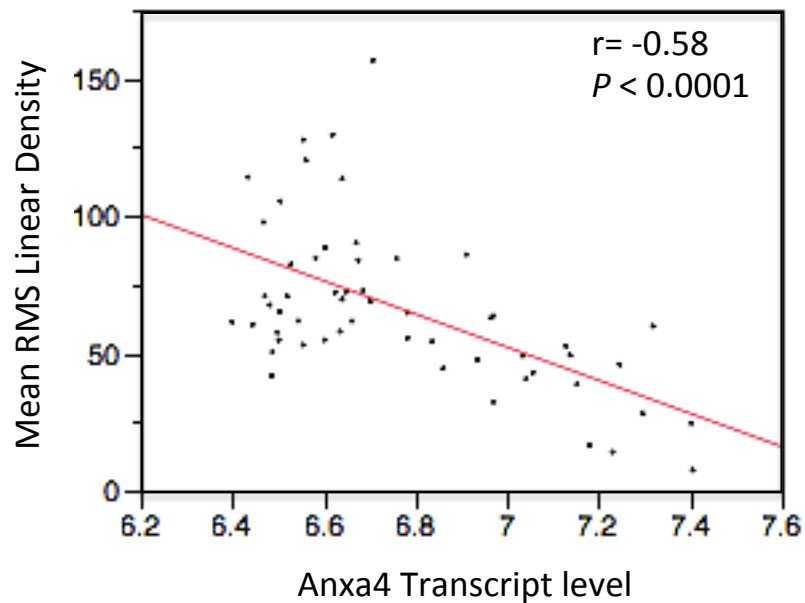**B**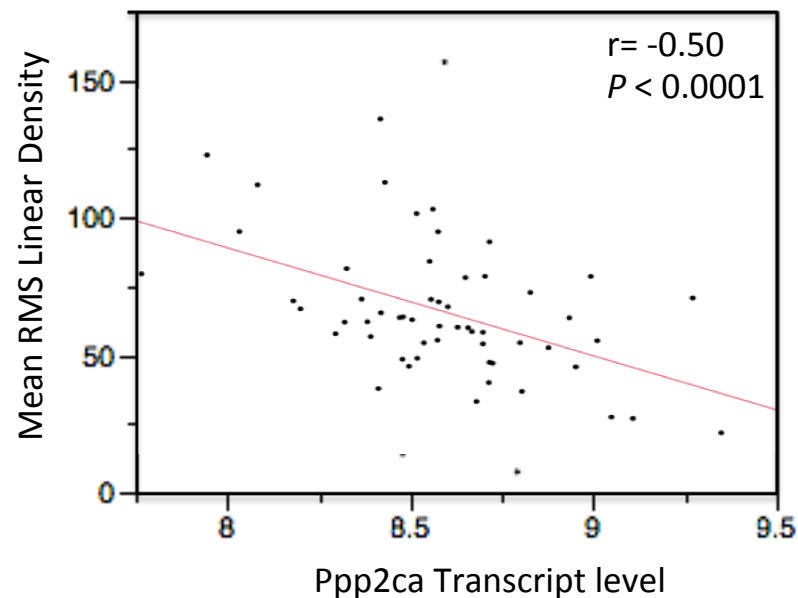**C**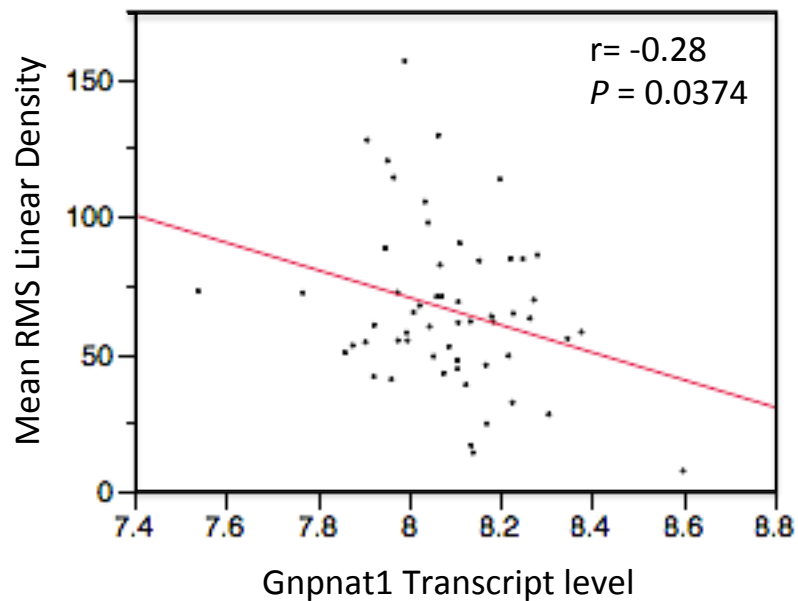**D**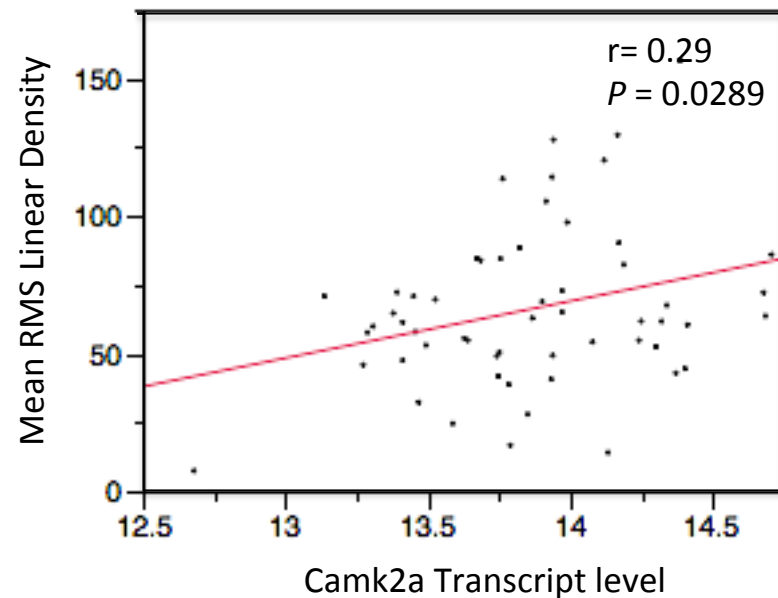

Supplement: Additional file 2 — Correlation between the transcript levels of candidate genes and RMS linear density. Inter-strain differences in the expression of Anxa4 (a candidate gene in the significant Chr 6 QTL interval), Ppp2ca (a candidate gene in the suggestive Chr 11 QTL interval), Gnpnat1 (a candidate gene in the suggestive Chr 14 QTL interval), and Camk2a (a candidate gene in the suggestive Chr 18 QTL) were observed in the hippocampi of different BXD RI strains. (A) Scatterplot of the Anxa4 transcript levels negatively correlated with the mean RMS linear density. (B) Scatterplot of the Ppp2ca transcript levels negatively correlated with the mean RMS linear density. (C) Scatterplot of the Gnpnat1 transcript levels negatively correlated with the mean RMS linear density. (D) Scatterplot of the Camk2a transcript levels positively correlated with the mean RMS linear density. Each dot represents a BXD strain. [file 1471-2164-15-206-S2.pdf]

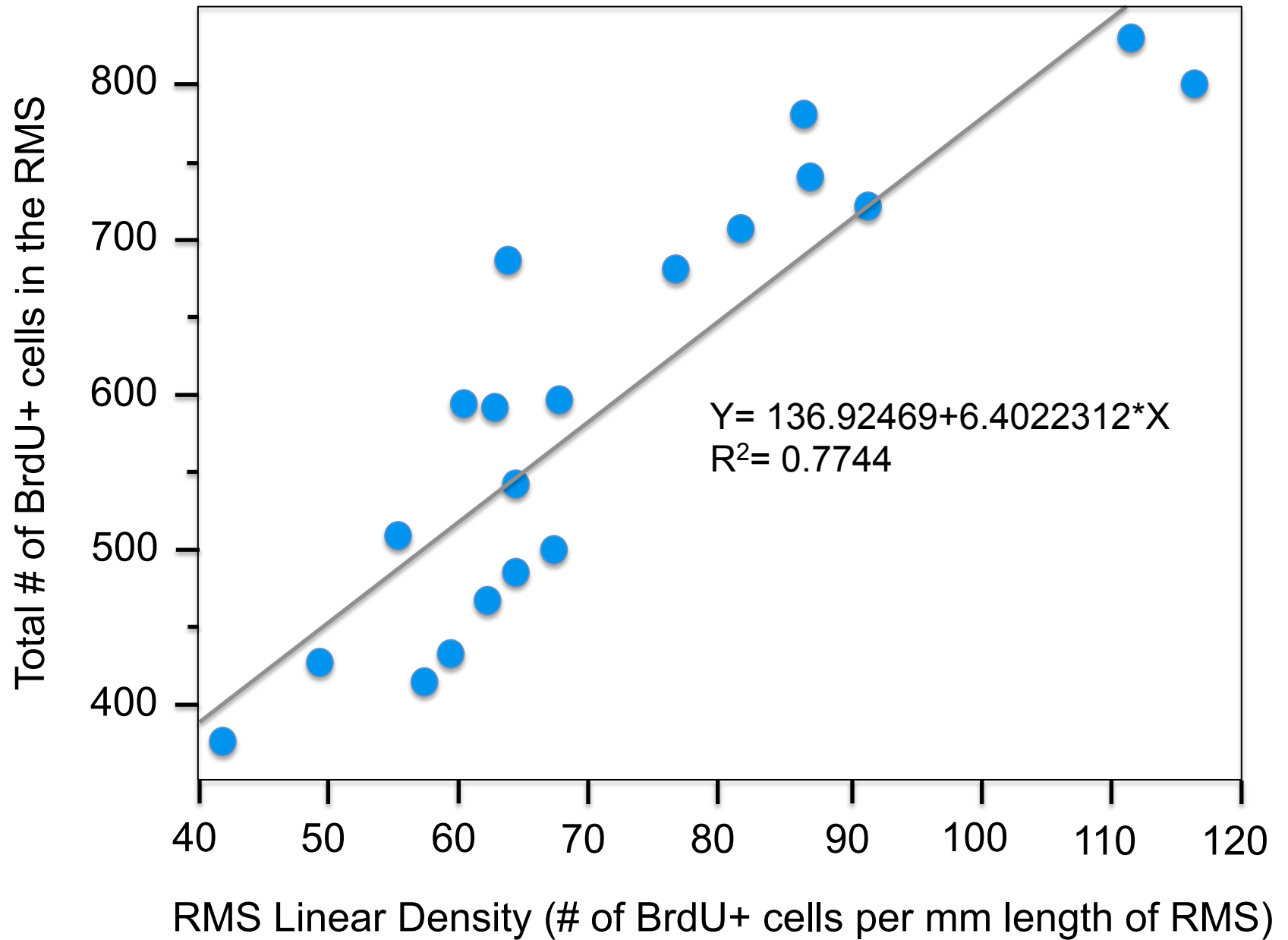

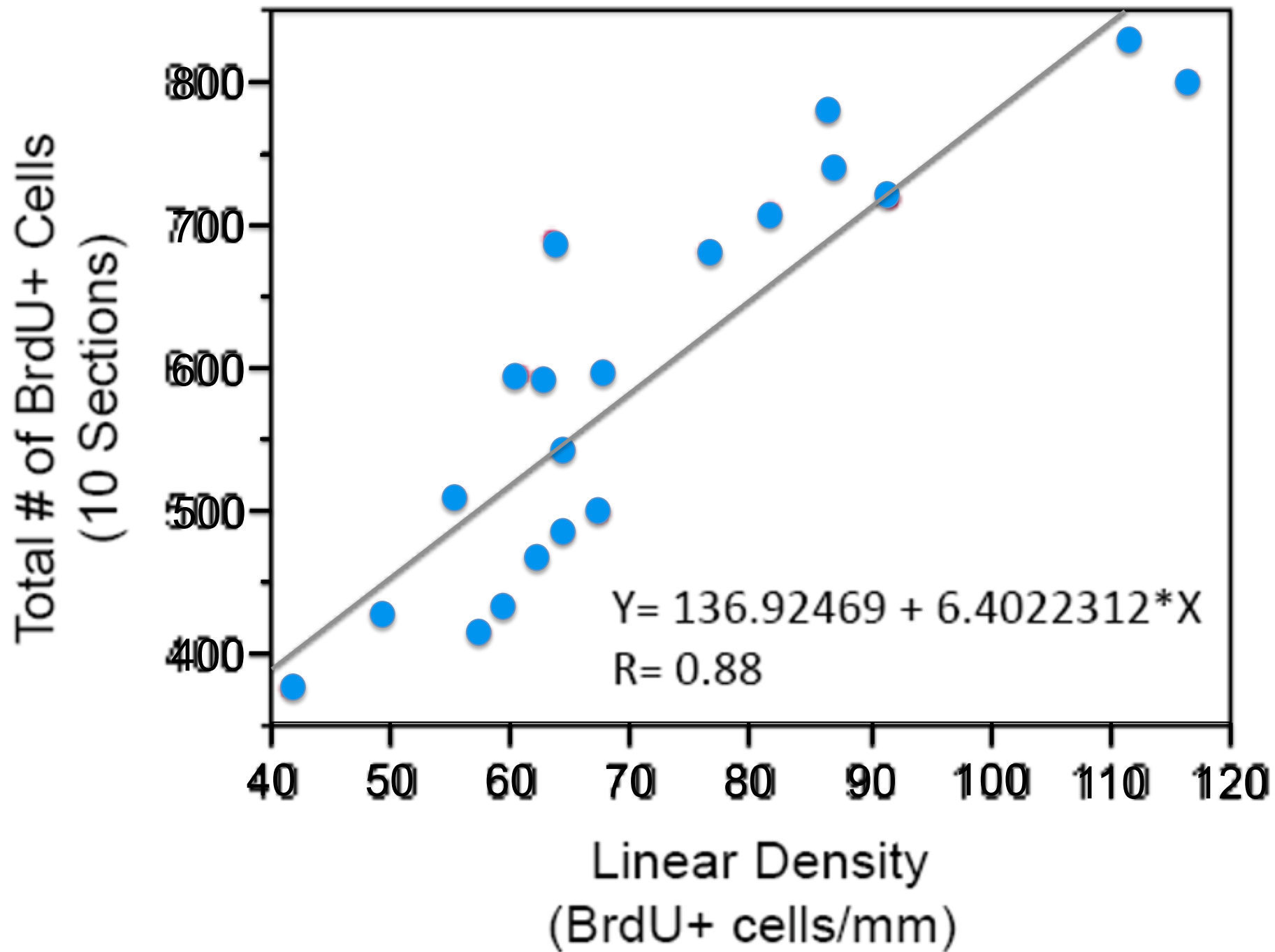

Supplement: Additional file 3 — Correlation between the RMS linear density and total number of proliferating NPCs in the RMS. RMS linear density (i.e. the number of BrdU + cells per mm length of RMS; x-axis) was determined using the single best-section quantification method, and it is significantly correlated with the total BrdU + cell counts (y-axis) which was determined from surveying every 10th section throughout the medial to lateral extent of the RMS (P < 0.0001). Each data point represents counts obtained from a randomly selected RI mouse. [file 1471-2164-15-206-S3.pdf]
